# New potential in the treatment of moderate to severe intrauterine adhesions: influencing factors for menstrual improvement

**DOI:** 10.3389/frph.2025.1608143

**Published:** 2025-09-08

**Authors:** Xin-Yue Zhang

**Affiliations:** Heilongjiang University of Chinese Medicine, Harbin, China

**Keywords:** intrauterine adhesions(IUA), menstrual improvement, hysteroscope, mental health, review, women

## Abstract

**Background:**

This article reviews the research progress in recent years on the influencing factors for menstrual improvement in patients with moderate to severe Intrauterine Adhesions. To improve the treatment effect, reduce the risk of re-adhesion, optimize the treatment plan, enhance patients' quality of life, and prevent infertility and miscarriage.

**Objective:**

Identify the factors that may affect menstrual improvement in patients with moderate to severe Intrauterine Adhesions.

**Method:**

An in-depth literature search was carried out on four databases to sort out the research results on the influencing factors of menstrual improvement in patients with moderate to severe Intrauterine Adhesions from 2014 to 2024.

**Findings:**

The review incorporated 61 papers and found that the influencing factors for menstrual improvement in patients with moderate to severe Intrauterine Adhesions involve: (1) The development of the Müllerian duct and the levels of Anti-Müllerian Hormone. (2) The interaction and dynamic changes between Mesenchymal Stem Cells and the endocrine system. (3) The impact of hysteroscopic surgery on the endometrium and menstrual improvement, including the effects of the operation method, frequency of implementation, and postoperative management of hysteroscopic surgery. (4) The role of psychological factors.

**Discussion:**

The results of this review highlight the factors influencing menstrual improvement in patients with moderate to severe Intrauterine Adhesions. However, the influencing factors of menstrual improvement are multifaceted and interrelated. Future research needs to further explore the interactions among these factors and how to optimize treatment plans to improve treatment outcomes.

## Graphical Abstract

**Figure:**
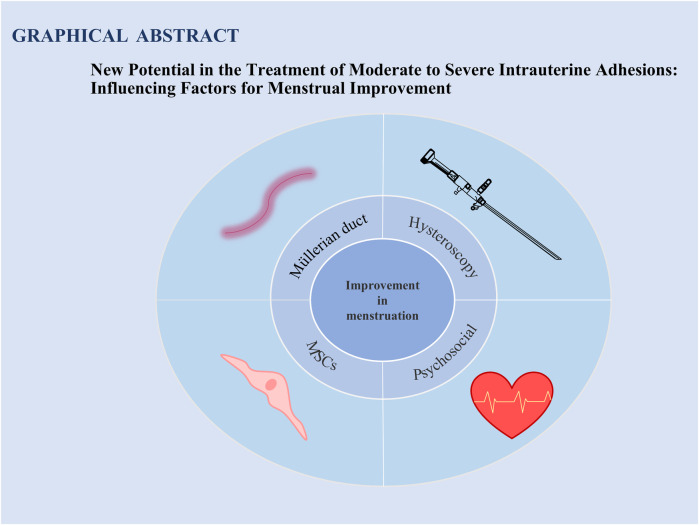

## 1 Introduction

IUA is an intrauterine lesion resulting from trauma and/or infection of the endometrial basal layer (1, 2). The pathogenesis of IUA remains incompletely understood. Nevertheless, it is commonly thought to be associated with the mutual adhesion between the uterine muscular walls following damage to the basal layer of the endometrium. This process encompasses three stages: the inflammatory stage, the tissue formation stage, and the tissue reconstruction stage (1). Given that the repair of the endometrium mostly involves incomplete regeneration, it eventually results in functional impairment and scar formation. The clinical manifestations of IUA patients mainly include reduced menstrual flow, amenorrhea, dysmenorrhea, infertility, and recurrent miscarriage. The severity of these symptoms is positively correlated with the degree of IUA (3). In severe cases, IUA may have a profound impact on a woman's fertility and mental health (2). Because of this, in recent years, numerous scholars have conducted extensive and in-depth research on the influencing factors of menstrual improvement in patients with moderate to severe IUA (Table 1) (4). This paper aims to review the latest progress of these studies, with the hope of providing more accurate professional guidance for clinical practice.

**Figure 1:**
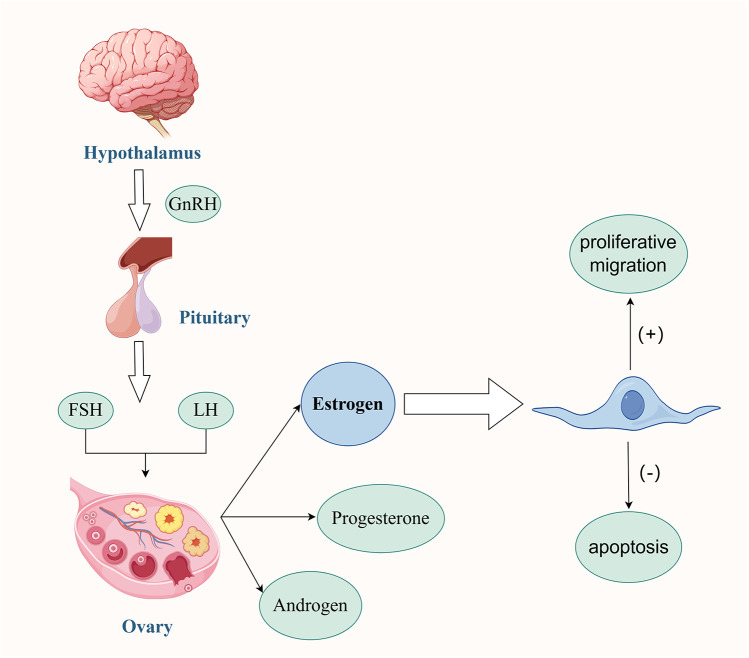
Interaction between MSCs and Estrogen. Under the regulatory mechanism of the human Hypothalamus-Pituitary-Ovary Gonadal Axis, the secreted Estrogen plays several key roles. On one hand, it can effectively promote the proliferation and migration of MSCs, enhancing their cell activity. On the other hand, it can also inhibit the apoptosis of MSCs themselves, maintaining the number of viable cells. Estrogen creates a microenvironment suitable for the survival of MSCs. In this environment, the two work together to promote the angiogenesis process in the damaged endometrial tissue. This angiogenesis effect further significantly improves the menstrual conditions of patients with moderate to severe IUA, contributing to the restoration of the normal physiological functions of the patients. Created with FigDraw.com.

**Figure 2:**
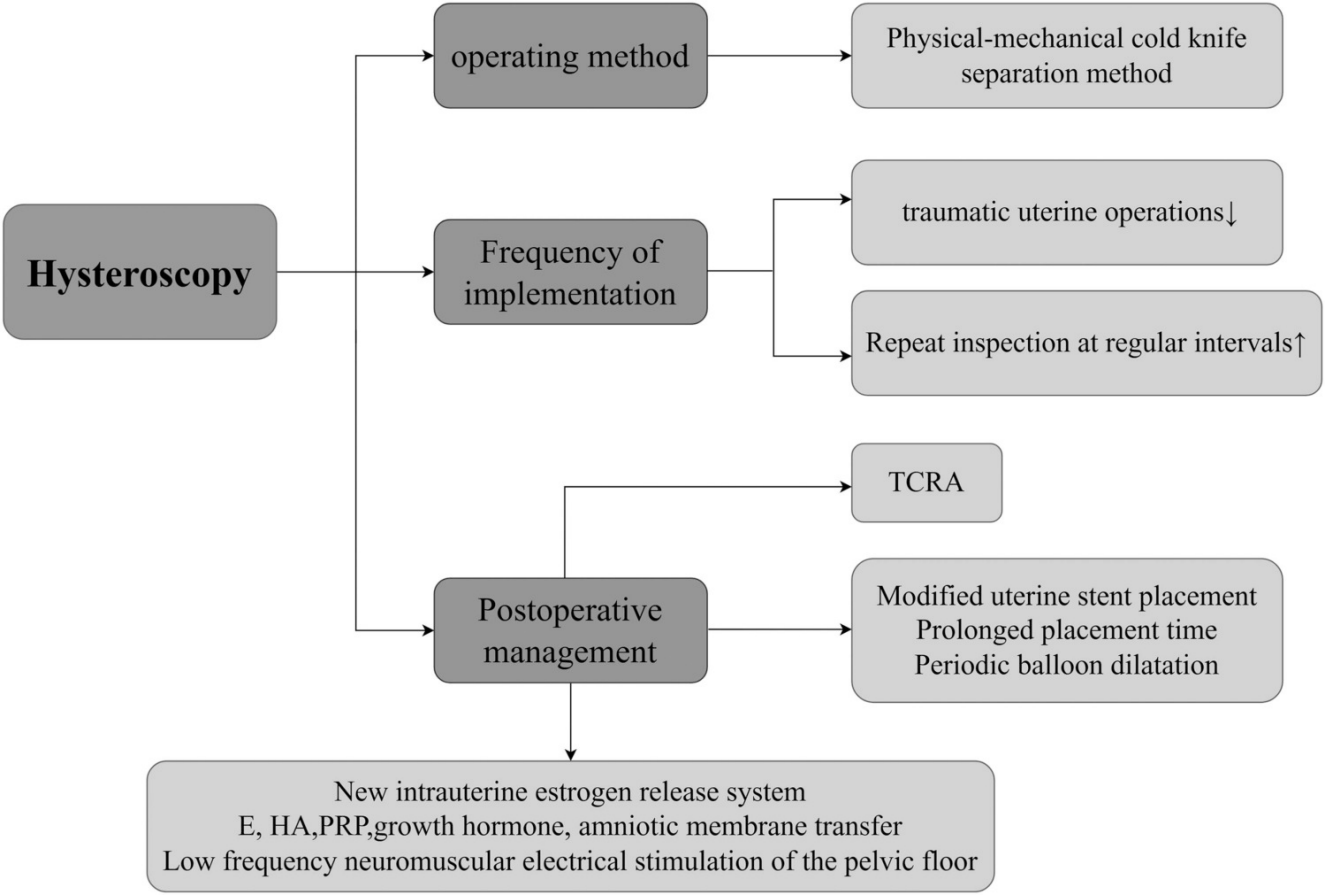
Approaches of hysteroscopic surgery to improve menstruation. This mainly involves the following three aspects: (1) Operation Method. At the level of the operation method, the physical-mechanical cold-knife separation method is the preferred choice. (2) Execution Frequency. Reducing the number of traumatic intrauterine operations during surgery and conducting regular hysteroscopic examinations after surgery jointly constitute a key link in ensuring the effectiveness of the surgery. (3) Postoperative Management, In terms of postoperative management, a modified uterine stent is adopted, and the placement time of the stent is appropriately extended to prevent intrauterine re-adhesion. Periodic balloon dilation is carried out to maintain the shape of the uterine cavity. Adjuvant artificial cycle therapy is used to regulate the endocrine level. Moreover, measures such as the application of Hyaluronic Acid, Platelet-Rich Plasma, pelvic floor low-frequency neuromuscular electrical stimulation, intrauterine perfusion of growth hormone, and amniotic membrane transplantation can promote the repair and regeneration of the endometrium from different perspectives. In patients with moderate to severe IUA, the comprehensive treatment approach centered around hysteroscopy is widely applied in clinical practice. Created with FigDraw.com.

**Table 1:** Classification and scoring criteria for intrauterine adhesions by the American fertility society.

Evaluation items	Description of project criteria	Score (points)
Adhesion scope	<1/3	1
	1/3–2/3	2
	>2/3	4
Adhesion type	Membranous	1
	Membranous + dense	2
	Dense	4
Menstrual type	Normalcy	0
	Oligomenorrhea	2
	Mmenorrhea	4

1 to 4 points indicate mild adhesions; 5 to 8 points indicate moderate adhesions; 9 to 12 points indicate severe adhesions.

## 2 Müllerian duct development and Anti-Müllerian hormone (AMH) levels

The Müllerian duct is the simplest basic duct and the cornerstone of the female reproductive tract. This structure consists of an epithelial lumen and the surrounding mesenchymal layer (5). As the fundamental structure of the female reproductive tract, the development of the Müllerian duct is crucial for the formation of the female reproductive system. The uterine morphological and functional abnormalities associated with Müllerian duct anomalies (6) increase the susceptibility of the endometrium to trauma. During the post-trauma repair process, they are more likely to trigger abnormal extracellular matrix deposition and tissue fibrosis, thus significantly elevating the risk of IUA. AMH, also known as Müllerian inhibiting substance (7), plays a very important role in regulating cell differentiation and inhibiting the development of the Müllerian duct (8). In the bodies of adult females, Follicle-Stimulating Hormone can stimulate the expression of AMH. AMH exerts an inhibitory effect by counteracting follicle-stimulating hormone, thus promoting the gradual regression of the Müllerian duct (9). AMH assesses the ovarian reserve function through the quantity and quality of ovarian follicles in women. Its level is positively correlated with the ovarian reserve function (10) but negatively correlated with age (11, 12). Therefore, among patients with moderate to severe IUA, the improvement in menstruation in young women is generally better than that in middle-aged and elderly women. This may be closely related to the good development of the Müllerian duct and the relatively high level of AMH in young women.

## 3 The interrelationship between mesenchymal stem cells (MSCs) and the endocrine system

MSCs are a type of multipotent stem cells, renowned for their self-renewal and multilineage differentiation potential (13). MSCs have a rich variety of sources, mainly including umbilical cord mesenchymal stem cells, bone marrow mesenchymal stem cells, adipose tissue-derived stromal cells, human amniotic mesenchymal stem cells, placental mesenchymal stem cells, etc. These are all hot topics in research and application (13). Some studies have shown that MSCs can not only promote the repair and regeneration of the endometrium through paracrine action (14) but also increase the vitality of the damaged endometrium via exosomes (15, 16), inhibit and even reverse the process of endometrial fibrosis (17, 18). The specific mechanisms of action may involve multiple aspects such as promoting angiogenesis (19), regulating the inflammatory response (20), and providing nutritional support. These characteristics endow MSCs with unique advantages and potential in the restoration of menstruation in IUA patients. In addition, MSCs can maintain the homeostasis of the uterine internal environment. The balance between self-differentiation and self-renewal of stem cells is crucial for the cyclic regeneration of the endometrium (13). Under physiological conditions, the human body is precisely regulated by the Hypothalamic-Pituitary-Ovarian-Gonadal Axis. As a result, the endometrium undergoes cyclic exfoliation and repair, demonstrating a high regenerative potential. Among them, the level of Estrogen is one of the key factors promoting the repair and regeneration of the endometrium. Some studies have confirmed (21) that the estrogen secreted by MSCs plays a decisive role in therapeutic applications. Estrogen not only promotes the proliferation and migration of MSCs (22) but also inhibits their apoptosis, providing a suitable microenvironment for the survival of MSCs (13). This interaction synergistically promotes angiogenesis in damaged tissues, thereby facilitating the repair and regeneration of the endometrium (23, 24). The endometrium is divided into the superficial functional layer and the deep basal layer. The integrity and continuity of the basal layer are the keys to the cyclic repair and regeneration of the endometrium (25). Some studies have found (26) that when the deep basal layer of the endometrium suffers severe damage, the vast majority of the basal layer is often replaced by a single-layer epithelium and fibrous tissue. This leads to difficulty in the regeneration of the functional layer, a decline in the endometrium's self-repair ability, and the endometrium being in an abnormal state. In this case, even if the Estrogen level in the body is high, due to the reduction in the number of Estrogen receptors or the saturation of their functions, the response to Estrogen stimulation weakens, resulting in an unsatisfactory treatment effect (27). Therefore, for patients with moderate to severe IUA, an adequate Estrogen level and an endometrial basal layer that has not suffered severe damage are two indispensable conditions for achieving menstrual improvement (Figure 1) (28).

## 4 Impact of hysteroscopic surgery on endometrium and menstrual improvement

With the continuous development and increasing maturity of hysteroscopic technology, the comprehensive treatment method mainly based on hysteroscopy is being increasingly widely applied in patients with moderate to severe IUA (1). Therefore, the impact of hysteroscopic surgery on endometrial and menstrual improvement has become a hot topic in academic and clinical practice.

Hysteroscopic technology (3) is an innovative technique in the field of minimally invasive gynecological diagnosis and treatment (29), its core advantage is in using fiber-source endoscopes for precise inspection and treatment of the uterine cavity. The main operating mechanism of this technology is to insert an endoscope with a light source into the uterine cavity via the vagina and cervix, enabling direct and clear observation of multiple aspects such as the shape of the uterine cavity, the condition of the endometrium, the presence of endometrial polyps, the distribution of abnormal blood vessels, and the state of the bilateral fallopian tube openings (30). Meanwhile, surgical instruments can be introduced through their working channel to perform operations like tissue cutting, separation, and electrocoagulation for hemostasis to treat various intrauterine diseases. Additionally, specimens can be accurately collected under direct vision for pathological examination, providing a reliable basis for diagnosis and treatment (31). Given that the fiber-optic and lens design of the hysteroscope is extremely precise, the damage it causes to the endometrium is minimal, and theoretically, it should not significantly affect a woman's menstrual function. During Transcervical Resection of Adhesion (TCRA) performed under hysteroscopy, the mechanical cold-knife technique is often used to physically separate the adhesive tissue in patients with moderate to severe IUA. Although this process may cause a certain degree of damage to the endometrium, after a period of recovery following the surgery, the endometrial function can return to normal completely, and it will not have a long-term impact on menstrual improvement (32). One study mentioned (33) that it conducted a detailed analysis of the outcomes of hysteroscopic surgery in patients with different degrees of IUA. The results showed that 100% of patients with severe IUA experienced an increase in menstrual volume after hysteroscopic surgery (including a return to normal levels). This result may be closely related to multiple factors such as Surgical Technique, Follow-up Frequency, Barrier Methods, Hormone Therapy, Biological Adjuncts, Innovations (e.g., estrogen stents, HA, PRP, and smart medicine). The following will explore these influencing factors in depth (Figure 2).

### 4.1 Surgical technique

As a refined minimally invasive technique, TCRA surgery has been widely proven to be a safe, effective, scientific, and feasible primary method for improving the postoperative menstrual function of patients with moderate to severe IUA (34). TCRA enables precise diagnosis of the location, extent, nature, and severity of IUA under direct visualization (4). It then selectively separates adhesive bands and excises scar-like adhesive tissues.The aim is to protect the remaining endometrium and restore the anatomical shape of the uterine cavity. Relatively speaking, the surgical safety is high. TCRA significantly improves the menstrual conditions of IUA patients. On one hand, it can eliminate the occurrence of IUA. On the other hand, it can minimize damage to the endometrium, thus facilitating the restoration of the uterine shape and function to the optimal state (35). According to the different instruments used during the operation, TCRA can be divided into two types: mechanical surgery and energy-based surgery, that is, the traditional cold-knife separation method and the hot-knife separation method. At the histological level, cold knife surgery is based on purely mechanical sharp cutting without thermal effects, with clear sections and no coagulation necrosis at the edges, which facilitates pathological evaluation and maximizes the protection of the endometrial basal layer, thus reducing the inflammatory response of the body and promoting endometrial regeneration and uterine cavity morphology restoration (36). In contrast, energy devices, which use a “thermal spreading band” to synchronize coagulation, may involve adjacent tissues and increase the risk of delayed injury. Meta-analysis showed (37) that bipolar vessel sealers reduced blood loss and operative time in laparoscopic hepatectomies, but in hysteroscopy, cold knife microsurgery was still better than plasma electrosurgery for improving menstrual abnormalities. plasma electrosurgery (35). Therefore, to more effectively improve the menstrual conditions of patients with moderate to severe IUA, the physical and mechanical cold-knife separation method in TCRA surgery should be regarded as the preferred surgical approach. Its scientific nature, safety, and effectiveness have been widely recognized (38). However, in the future, there is still a need for local spraying or infiltration of hemostatic agents in the operative field, combined with microenergetic punctual coagulation and real-time visualization and navigation, which can significantly reduce bleeding and shorten the operation time of cold knife surgery without introducing obvious thermal damage, to better applying cold knife surgery to a wide range of clinical work.

### 4.2 Follow-up frequency

In the field of hysteroscopic surgery, existing research has pointed out (39) that an increase in the frequency of traumatic intrauterine operations during surgery, especially multiple repeated operations in a short period, may significantly increase the risk of postoperative infection (39). Based on this, it is of particular importance to minimize the number of traumatic intrauterine procedures during surgery and to actively adopt preventive measures after surgery to reduce the risk of infection. In the research of Yang Jianghua and other scholars (40), by strengthening hysteroscopic reexamination every month after surgery and combining with the comprehensive preventive treatment of injecting sodium hyaluronate gel, estradiol valerate gel, and periodic progesterone into the uterine cavity, significant clinical effects have been demonstrated. On one hand, this research achieves local hemostasis by inhibiting the activation and aggregation of inflammatory cells, thus reducing the inflammatory exudation of the wound surface. On the other hand, it can significantly suppress the generation of fibroblasts and reduce the hyperplasia of collagen fibers, thereby reducing the formation of surgical scars. This can effectively prevent re-adhesion in IUA patients, thereby shortening the recovery time of the endometrium. The research results indicate (40) that this comprehensive treatment strategy has enabled the menstrual improvement rate of patients with moderate to severe IUA to reach 94.3% after hysteroscopic surgery. This finding shows a statistically significant difference, providing a new perspective and method in the field of intrauterine adhesion treatment. In addition, Wang X, Duan H (41) also reported a remarkable finding in their research: the cure rate of TCRA secondary treatment is as high as 100%. This result may suggest that, for all the patients involved in the study, after the second-round treatment, the intrauterine adhesions were completely resolved, their menstrual functions returned to normal, and no recurrence of adhesions was observed. In short, for patients with moderate to severe IUA undergoing hysteroscopic surgery, minimizing traumatic intrauterine procedures during the operation and conducting regular hysteroscopic examinations after the surgery are the key factors in ensuring surgical effectiveness and patient recovery.

### 4.3 Barrier methods

Precise hysteroscopic placement of modified uterine stents has been confirmed by several studies to synchronize postoperative menstrual flow and endometrial thickness (42), initially establishing its central position in the functional reconstruction of moderate-to-severe IUA. On this basis, moderately prolonged balloon stent retention or cyclic balloon dilatation (43, 44) can continue to isolate the trauma and stabilize the uterine cavity morphology in the critical postoperative window, thereby depressing the recurrence rate of IUA in the long term and consistently amplifying menstrual benefit (45), providing a direct and quantifiable evidence-based barrier strategy for moderate-to-severe IUA.

### 4.4 Hormone therapy

Artificial cycles constructed with high-dose transdermal estradiol gel (6 mg/d) (46) in combination with vaginal estradiol (47) and high-dose transdermal estradiol gel (6 mg/d) in combination with vaginal estrogens can safely and effectively remodel the endothelium after moderate-to-severe IUA: the improvement rate of menstruation was 67% (46), and was comparable to that of the oral formulation. Transdermal and transvaginal administration bypasses the first-pass elimination effect in the liver and has a lower risk of thrombosis, providing a highly effective and safe exogenous estrogen regimen for postoperative endothelial repair.

### 4.5 Biological adjunct

In patients with moderate-to-severe IUA who had failed previous hysteroscopy, amniotic membrane transplantation (48) immediately after microscopic separation induced crawling regeneration of the basement membrane and rapid reconstruction of the functional layer; G-CSF intrauterine perfusion was initiated within the seventh postoperative day, and the sequential regimen of combined growth hormone and growth factor could synchronize the promotion of neovascularization, cell proliferation, and the inhibition of apoptosis (49). Multicenter data showed (49) that this strategy can thicken the endothelium by an average of 2.36 mm, significantly reduce intraoperative bleeding and shorten the menstrual cycle, and provide a highly efficient and reproducible sequential pathway for functional reconstruction.

### 4.6 Innovations (e.g., estrogen stents, HA, PRP, and smart medicine)

Multicenter RCT demonstrated (50) that immediate postoperative insertion of the new intrauterine extended-release estrogen system (IERS) could reduce the recurrence rate of moderate-to-severe IUA to <10%, which was significantly better than that of the traditional balloon, while the local steady release of E₂ improved the menstrual flow score by ≥2 points in 72% of the patients in 3 months, realizing the dual benefit of “mechanical barrier + high estrogen concentration”.Sodium hyaluronate gel (Hyaluronic Acid, HA) (51)forms a biodegradable protective layer on the trauma and reduces bleeding, exudation, and inflammation, thus systematically decreasing the incidence and severity of adhesions (52). Intrauterine injections of platelet-rich plasma (PRP) (53–55) improve the prognosis of fertility by concentrating growth factors and anti-inflammatory factors, synergizing with hormones and physical barriers to rapidly rebuild the basal layer and inhibit fibrosis. In addition, low-frequency neuromuscular stimulation of the pelvic floor, as an intelligent means of rehabilitation, further promotes local vascularization and enhances the rate of menstrual recovery (56). Although there is a consensus that the more severe the IUA, the worse the prognosis, multimodal combined interventions are gradually reversing this dilemma (39). Therefore, 7 d postoperative balloon or 1–2 months IUD with mechanical barrier rapidly depresses the AFS score, the balloon is slightly better in reconstructing the uterine cavity morphology, and the IUD is additional anti-inflammatory with copper ions; on top of this (42–45), the self-crosslinked HA gel resides in semi-solid state for 7–14 d, which is confirmed by Meta-analysis to reduce the rate of moderate-to-severe adhesion recurrence and to achieve zero adverse effects (51, 52). The synergistic effect of PRP intrauterine administration further amplified the pro-restorative effects of HA and estrogen scaffolds, and microscopic endothelial injections reduced AFS scores and improved menstrual flow rates compared with perfusion alone (53–55). Taking into account the available evidence, the combination of “mechanical barrier + HA + PRP” can be recommended as the first-line optimization strategy after moderate-to-severe IUA.

## 5 Psychological factors

Psychological factors play a pivotal role in the improvement of menstrual function in patients with moderate to severe IUA. Due to the long treatment cycles, complex treatment procedures, uncertain treatment efficacy, heavy economic burden, and high recurrence rate of adhesions that IUA patients face, these patients often experience negative emotions such as anxiety and depression (57). Therefore, it is particularly important to develop and implement targeted psychological intervention measures to alleviate patients' psychological burden and improve their quality of life (58). Research shows (57) that a harmonious partner relationship can encourage both spouses to participate in and cooperate during the treatment of IUA, which can effectively relieve the patient's psychological stress. A study by Yuqing Chen, Huan Yang, et al. has found (59) that negative emotions may interfere with sex hormone levels, affect the repair of the endometrium, and thus influence the improvement of menstruation. Mindfulness-Based Stress Reduction, as a method of psychological self-regulation training, has been proven to significantly improve negative emotions such as anxiety and depression in IUA patients during treatment by enhancing the state of mindfulness and adjusting the cognitive level, thus improving their quality of life. Si Jingge and Wang Sha also pointed out that disease-specific follow-up can provide effective guidance and supervision for IUA patients, especially in terms of postoperative adjuvant drug therapy. It can enhance patients' awareness and attention to the disease, avoid unfavorable factors, and standardize subsequent treatment plans. As a result, patients with IUA who receive disease-specific follow-up tend to experience a shorter time to menstrual resumption, a longer menstrual period, and better improvement in menstrual flow (60). In conclusion, psychological factors play a crucial role in the diagnosis and treatment of moderate to severe IUA. The emotional changes in patients are not only triggered by the occurrence of IUA but also, in turn, affect the treatment outcome of IUA (61).

## 6 Conclusions

In summary, in the research of the past decade, significant progress has been made in studies on improving menstruation in patients with moderate to severe IUA. This progress is not limited to the common clinical manifestations of IUA patients in traditional cognition, such as reduced menstruation or amenorrhea. The current research horizons are constantly expanding, covering but not limited to the following key areas: exploring the association between Müllerian duct development and AMH levels, the interaction and dynamic changes between MSCs and the endocrine system, and the impact of hysteroscopic surgery on the improvement of the endometrium and menstruation, and the crucial role of psychological factors in the treatment process. These multi-dimensional influencing factors jointly act on the process of improving the menstrual function of patients with moderate to severe IUA. Thus, in young patients with moderate-to-severe IUA, good restoration of menstrual patterns is generally achieved, even if the surgical and subsequent intervention regimen tends to be simplified, because of the maturation of the mullerian ducts, the relatively high levels of AMH and E, and the limited damage to the basal layer of the endothelium. The influencing factors for improving menstruation in patients with moderate to severe IUA involve multiple fields such as biology, endocrinology, and psychology. Considering these factors comprehensively and formulating individualized treatment plans are of great clinical significance in many aspects, including improving treatment efficacy, reducing the risk of re-adhesion, optimizing treatment regimens, enhancing the quality of life of patients, and preventing infertility and miscarriage. However, it cannot be ignored that the research on the influencing factors of menstrual improvement in patients with moderate to severe IUA still faces many challenges. At present, a clear theoretical framework has not been formed regarding the specific directions for improving menstrual function, which restricts the prediction and optimization of treatment effects. Additionally, there is a lack of adequate literature and data to support research on the effects of Müllerian duct development and AMH content on the improvement of menstruation, indicating that further research is needed to fill this knowledge gap. Thus, for patients with moderate to severe IUA, the influencing factors of menstrual improvement are multifaceted and interrelated. Future research needs to further explore the interactions among these factors and how to optimize treatment plans to improve treatment outcomes. At the same time, focusing on the development of individualized treatment strategies is also a key direction for enhancing treatment effectiveness.
